# Functional redundancy between *trans*-Golgi network SNARE family members in *Arabidopsis thaliana*

**DOI:** 10.1186/1471-2091-14-22

**Published:** 2013-09-11

**Authors:** Sang-Jin Kim, Diane C Bassham

**Affiliations:** 1 Department of Genetics, Development and Cell Biology, Iowa State University, Ames, IA 50011, USA; 2 Interdepartmental Genetics Program, Iowa State University, Ames, IA 50011, USA; 3 Plant Sciences Institute, Iowa State University, Ames, IA 50011, USA; 4 Current address: Great Lakes Bioenergy Research Center, Michigan State University, East Lansing, MI 48824, USA

**Keywords:** Membrane fusion, SNARE, *Trans*-Golgi network, Vesicle trafficking

## Abstract

**Background:**

Vesicle fusion is an essential process for maintaining the structure and function of the endomembrane system. Fusion is mediated by t-SNARE (soluble *N*-ethylmaleimide-sensitive factor attachment protein receptor) fusion proteins on the target membrane and v-SNAREs on the vesicle membrane; v-and t-SNAREs interact with each other, driving vesicle fusion with the target membrane. The *Arabidopsis thaliana trans*-Golgi network resident SNAREs SYP41 and VTI12, along with YKT61/62, have been shown to function in vesicle fusion *in vitro*, consistent with immunoprecipitation results showing their interaction in Arabidopsis cell extracts. Conflicting published results have indicated that SYP4 family members are either functionally redundant or have distinct and essential functions; the reason for this discrepancy is unclear.

**Results:**

Here we used a proteoliposome fusion assay to demonstrate that SYP42 and SYP43 can substitute for SYP41 in driving lipid mixing, providing support for functional overlap between family members. Previous reports have also suggested that VTI11 and VTI12 SNAREs show partial overlap in function, despite having mostly distinct localizations and binding partners. We show that VTI11 can substitute for VTI12 in *in vitro* lipid mixing reactions, providing molecular support for the genetic evidence for partial functional redundancy *in vivo*.

**Conclusions:**

Our data provide biochemical evidence for functional overlap in membrane fusion between members of the SYP4 or VTI1 SNARE groups, supporting previous genetic data suggesting redundancy.

## Background

The endomembrane system in plants, consisting of the endoplasmic reticulum, Golgi apparatus, *trans*-Golgi network (TGN), prevacuolar compartment (PVC), vacuole and endosomes, has important roles throughout development, in responses to stress conditions and in defense responses [1-3]. Transport between organelles of the endomembrane system is mediated by transport vesicles delivering appropriate proteins, lipids and polysaccharides. The correct trafficking of vesicles requires a number of proteins that function in processes from vesicle budding to vesicle fusion [4-6].

Soluble *N*-ethylmaleimide-sensitive factor attachment protein receptor (SNARE) proteins have a central role in vesicle trafficking in the recognition and fusion between vesicle and target membranes [7,8]. SNAREs have a coiled-coil domain which interacts with other SNAREs and typically have a C-terminal integral membrane domain for anchoring into the membrane. There are two functional types of SNAREs; v-SNAREs are inserted into the vesicular membrane, while t-SNAREs are located on the target membrane. SNAREs can be divided into 4 classes, Qa, Qb, Qc and R, depending on the presence of a conserved Q or R residue in their coiled-coil domain. Q-type SNAREs are usually located on the target membrane and R-type SNAREs are typically on the vesicular membrane [9]. In general, two or three t-SNARE polypeptides form a *cis*-SNARE complex on the target membrane, which interacts with a v-SNARE on an incoming vesicle via their coiled-coil domains, forming a four-helix *trans*-SNARE complex [10]. This *trans*-SNARE complex allows the vesicle to fuse with its target membrane and release its cargo. The *trans*-SNARE complex is typically formed only by the correct combination of v-and t-SNAREs, which is one mechanism for providing fusion specificity [11]. The requirements for vesicle fusion have been studied extensively, and it has been demonstrated that SNARE complex formation is sufficient to drive membrane fusion in an *in vitro* proteoliposome fusion assay, suggesting that the SNAREs themselves form the core of the membrane fusion machinery [12-17].

The Arabidopsis TGN contains the SYP4 family of closely-related SNAREs, which has 3 members, SYP41, SYP42 and SYP43 [18,19]. SYP41 and SYP42 each interact with the t-SNARE SYP61 and v-SNARE VTI12 in addition to the SM (Sec1/Munc18) protein VPS45, a potential regulator of vesicle fusion [18,20,21]. There have been conflicting reports regarding the possible functional redundancy of the SYP4 family members. SYP41 and SYP42 were originally reported to be essential proteins in Arabidopsis, with *syp41* and *syp42* knockout single mutations being gametophyte lethal [22], suggesting that each SYP4 family member had a distinct function. However, a more recent study found that members of this family had redundant or overlapping functions, with no or only subtle phenotypes for the single mutants but lethality for the *syp41syp42syp43* triple mutant. Vacuolar and secretory trafficking was defective in a *syp42syp43* double mutant, suggesting a function of the SYP4 family in multiple trafficking pathways [23]. The reason for the discrepancies between these two studies is not clear.

The *VTI1* SNARE family consists of four genes (*VTI11, VTI12, VTI13* and *VTI14*) in Arabidopsis but only *VTI11* and *VTI12* are expressed at significant levels [19,24-26]. VTI11 and VTI12 have high amino acid sequence identity (60%), but they function in different trafficking pathways; VTI11 is involved in trafficking to the lytic vacuole, while VTI12 is involved in trafficking of storage proteins [27]. Mutant phenotypes of VTI11 and VTI12 are also distinct. A *vti11* mutant shows defects in shoot gravitropism, whereas a *vti12* mutant is defective in the autophagy pathway and a *vti11/vti12* double mutant is lethal [26,28]. Although VTI11 and VTI12 have different functions in vacuolar trafficking, overexpressed VTI12 or a mutant of VTI12 that changes its specificity can substitute for VTI11 in the *vti11* mutant [29], and VTI11 is also able to interact with SYP41 and SYP42 when VTI12 is not available [26]. These genetic studies suggest that VTI11 may be able to functionally substitute for VTI12 in the SYP4 SNARE complex to drive vesicle fusion, although this has not been addressed directly.

Previously, either SYP41 or SYP61, together with VTI12 and the multifunctional SNARE YKT61/62, were found to be sufficient to drive lipid mixing of liposomes *in vitro*[15]. In yeast and mammalian cells, Ykt6 is involved in vacuolar trafficking and recycling from endosomes to the TGN by interacting with Vti1p [30-33]. In Arabidopsis, there are two proteins related to yeast Ykt6, and both of them are able to drive membrane fusion with SYP41 and VTI12 *in vitro*, suggesting that they may be functionally redundant [15].The requirement for only three SNARE proteins in the fusion reaction suggests that either a three-helix bundle may be formed at the Arabidopsis TGN or, more likely, two molecules of one component may be required to make a four-helix bundle.

Here, we use *in vitro* liposome lipid mixing assays to address two questions relating to TGN SNARE redundancy: can SYP42 and/or SYP43 substitute for SYP41 in the *in vitro* lipid mixing assays, thus providing further evidence to distinguish between overlapping vs. distinct functions of these SNAREs?; and can VTI11 also drive fusion in combination with SYP4 SNAREs and YKT61/62, providing biochemical evidence that VTI11 may be able to functionally substitute for VTI12 in the SYP4 SNARE complex when VTI12 is absent, thus supporting the genetic studies [29]? We show that SYP42 or SYP43 reconstituted into vesicles are also able to drive lipid mixing with VTI12-containing vesicles, supporting the idea of functional redundancy between these SNAREs. In addition, VTI11-containing vesicles are able to fuse with vesicles containing a SYP4 family member, indicating that functional overlap between VTI11 and VTI12 is mediated by interaction and fusion activity with SYP4 family proteins.

## Results

### Expression of recombinant SNAREs in *Escherichia coli*

**Figure 1:**
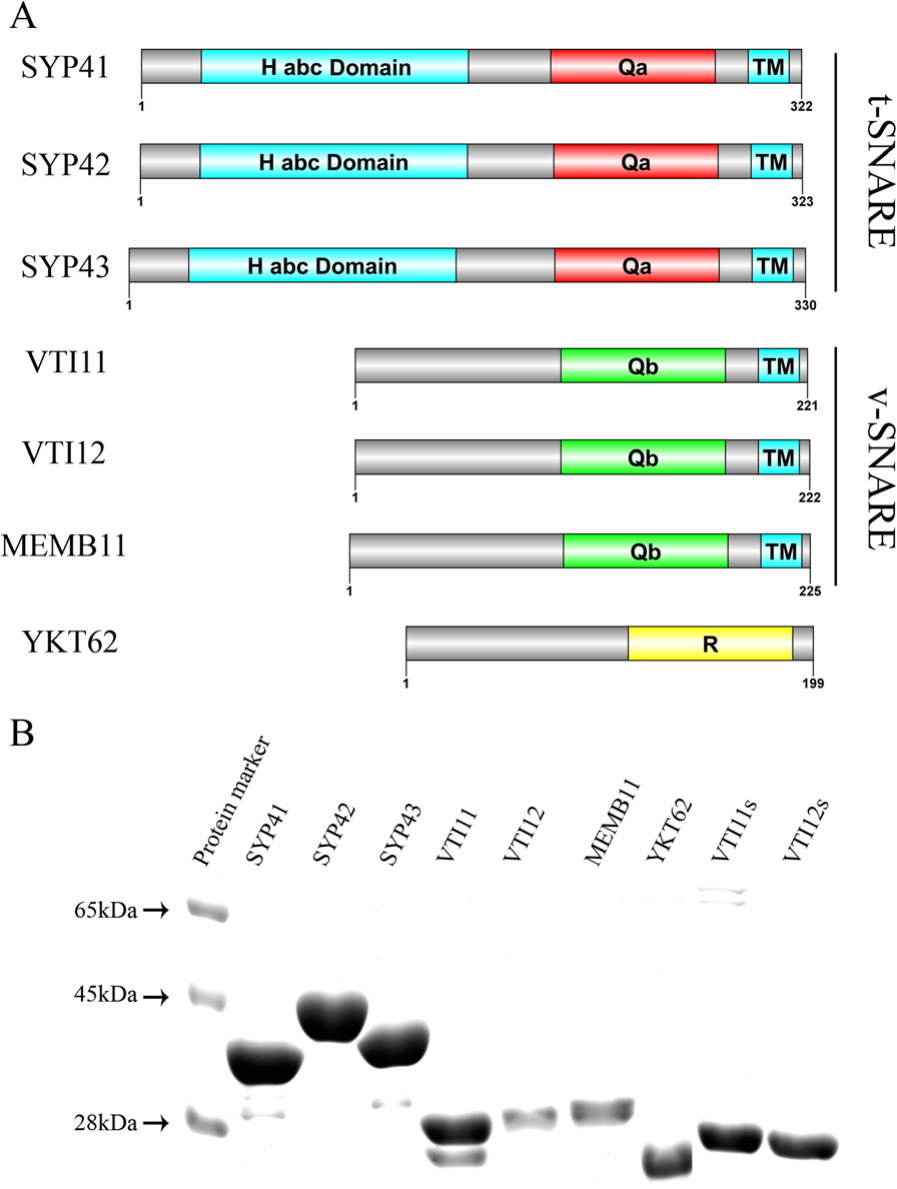
**Expression of recombinant proteins for lipid mixing assays. A**. Schematic diagram of proteins used in this study [34] showing their domain structure. Habc, the N-terminal domain found in Qa-type SNAREs. TM, transmembrane domain. **B**. Expression and purification of proteins. His-tagged recombinant proteins as indicated were expressed in *E.coli* and purified using Ni-NTA resin. Ten μg each of the purified proteins were separated by SDS-PAGE and stained using Coomassie Blue. MW markers are indicated at left.

SNAREs used in this study are shown schematically in Figure 1A. Recombinant proteins were expressed in *E. coli* and purified via their N-terminal His_6_ tag using Ni-NTA resin. Purified proteins were separated by SDS-PAGE and stained with Coomassie Blue. The purified proteins migrated at their expected MW with the exception of SYP42 (Figure 1B). The predicted size of SYP42 is approximately 36 kDa, but recombinant SYP42 migrated at around 45 kDa, presumably due to structural hindrance during migration in the gel (Figure 1B). This size difference was also observed in a previous study of epitope-tagged SYP42 in transgenic Arabidopsis plants [18].

### SYP42 and SYP43 can also drive liposome fusion

**Figure 2:**
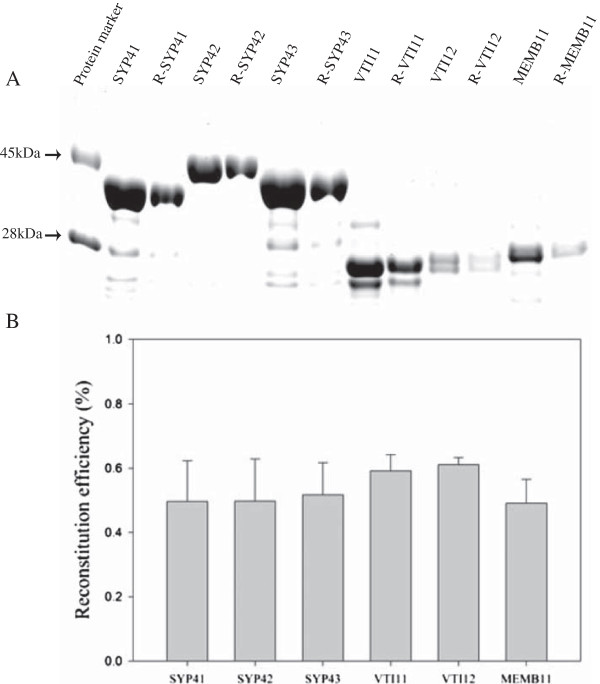
**Reconstitution of recombinant proteins into liposomes. A**. Recombinant proteins were reconstituted into liposomes in the presence of detergent (Triton X-100 or n-octylglucoside), followed by dialysis to remove detergents after reconstitution. The amount of each protein before and after reconstitution was analyzed by SDS-PAGE and Coomassie Blue staining to determine the reconstitution efficiency. Prefix R- stands for reconstituted proteins. **B**. The average reconstitution efficiency was obtained from three independent experiments by densitometry of gels. The average reconstitution efficiency was ~50% for all proteins. Error bars represent standard deviation (n = 3).

Previously, proteoliposome fusion assays using SYP41, VTI12 and YKT62 demonstrated that these proteins were sufficient to drive full lipid mixing between liposomes, as a proxy for vesicle fusion [15]. Conflicting reports in the literature have suggested that the SYP4 family SNAREs have either distinct [22] or redundant [23] functions. To further address this issue, we tested whether other SYP4 family proteins can substitute for SYP41 in driving lipid mixing in combination with VTI12 and YKT62. Recombinant SYP41, SYP42 or SYP43 was reconstituted into phospholipid vesicles mimicking plant membrane composition (acceptor vesicles) containing 60 mol% 1,2-dioleoyl-sn-glycero-3-phosphocholine (DOPC), 20 mol% 1-palmitoyl-2-oleoyl-sn-glycero-3-(phospho-rac-(1-glycerol)) (POPG) and 20 mol% 1-palmitoyl-2-oleoyl-sn-glycero-3-phospho-ethanolamine (POPE) using a Bio-beads/dialysis method [14]. VTI12 was reconstituted into separate vesicles (donor vesicles) of the same lipid composition, except for the addition of 1.5 mol% each of N-(7-nitro-2,1,3,benzoxadiazol-4-yl) (NBD) and rhodamine-labeled fluorescent lipids (NBD-PE and rhodamine-PE) replacing an equal amount of DOPC. The efficiency of reconstitution of proteins into liposomes was analyzed by SDS-PAGE and staining with Coomassie Blue, and the reconstitution efficiency as determined by densitometry was around 50-60% for each protein (Figure 2).

The basis for the *in vitro* liposome lipid mixing assay is loss of fluorescence resonance energy transfer (FRET) due to dilution of the fluorescent lipids. Donor vesicles contain NBD and rhodamine-labeled fluorescent lipids, whereas acceptor vesicles are unlabeled. NBD fluorescence in donor vesicles is quenched by rhodamine resulting in very low initial fluorescence. When donor and acceptor vesicle membranes fuse, dilution of the fluorescent lipids leads to a decrease in NBD fluorescence quenching, causing an increased NBD fluorescence which can be monitored by fluorescence spectrophotometry.

**Figure 3:**
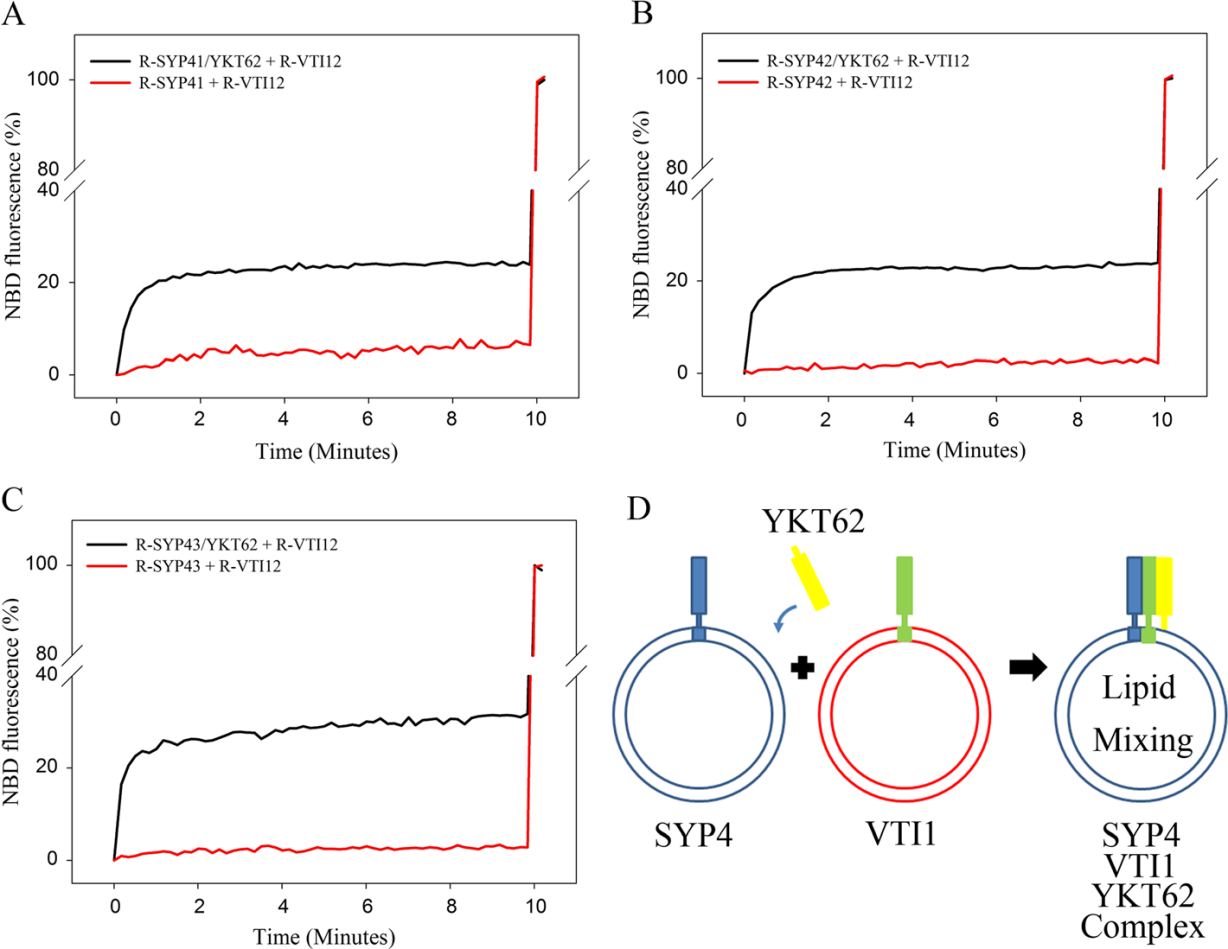
**SYP42 and SYP43 can drive liposome fusion.** Purified SYP41 **(A)**, SYP42 **(B)**, or SYP43 **(C)** was incorporated into acceptor vesicles (non-fluorescent vesicles), and VTI12 was reconstituted into donor vesicles containing fluorescent lipids. Membrane fusion with (black) or without (red) YKT62 was measured by monitoring NBD fluorescence with excitation wavelength of 460 nm and emission wavelength of 530 nm. The fluorescence was recorded every ten seconds for 10 min. The maximal fluorescence intensity (MFI) was obtained by adding 1 μl of 10% (v/v) Triton X-100, and recorded fluorescence measurements were normalized to the MFI. **D**. Schematic diagram of the assays shown in Figures 3 and 4.

Acceptor vesicles containing SYP41, SYP42 or SYP43 and donor vesicles containing VTI12 were mixed together with the soluble form of YKT62, which is normally present as a lipid-modified form for membrane association in yeast and lacks a transmembrane domain (Figure 3D). This led to a rapid increase in NBD fluorescence due to dilution of the fluorescent lipids caused by membrane fusion (Figure 3A-C). These data indicate that SYP41, SYP42 and SYP43 can all drive lipid mixing of liposomes, and that SYP42 and SYP43 can function with VTI12 and YKT62 in SNARE complexes *in vitro*, as previously suggested by their co-immunoprecipitation [18]. No fusion was observed when YKT62 was omitted from each fusion reaction, suggesting YKT62 is also required for membrane fusion with SYP4 and VTI1 family members and that lipid mixing is dependent on the presence of the SNAREs (Figure 3). This result provides support for the idea of functional overlap between the SYP4 SNAREs in vesicle fusion.

### VTI11 can drive membrane lipid mixing in combination with SYP4 family SNAREs

In Arabidopsis, four genes (*VTI11, VTI12, VTI13* and *VTI14*) comprise the VTI1 family, with *VTI14* only expressed in Arabidopsis suspension cells [19]. VTI11 and VTI12 are v-SNAREs localized to the PVC and TGN, respectively [35]. VTI11 and VTI12 were previously shown to compensate for each other in each single knockout mutant, as a *vti11vti12* double mutant is embryo lethal, suggesting they are at least partially functionally redundant [26]. Niihama et al. [29] found that expression of *VTI12* was increased in a *vti11* mutant, and a single amino acid substitution in VTI12 which changes the cellular localization of VTI12 to the PVC suppressed the *vti11* mutant phenotype. We hypothesize that VTI11, in addition to VTI12, is also able to mediate vesicle fusion in combination with members of the SYP4 family and that the specificity of SNARE complex formation *in vivo* is due at least in part to the distinct localization of VTI11 and VTI12.

**Figure 4:**
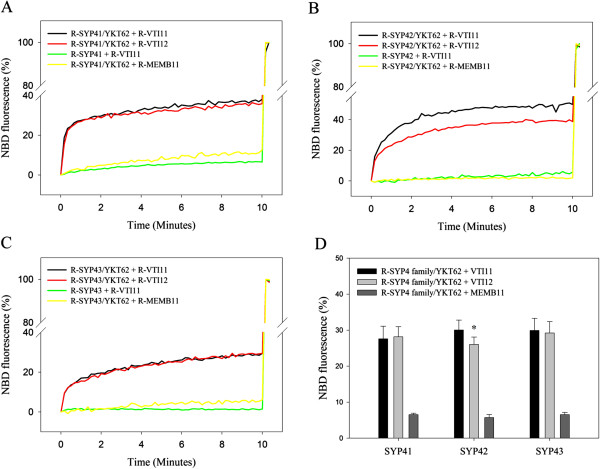
**SYP4 family members and VTI11 can function together in membrane fusion. A**-**C**. Purified SYP41 **(A)**, SYP42 **(B)** or SYP43 **(C)** were incorporated into acceptor vesicles, and VTI11, VTI12 or MEMB11 were reconstituted into donor vesicles. Mixing of acceptor vesicles and donor vesicles was measured as in Figure 3. The change in NBD fluorescence was monitored after mixing each R-SYP4 family and R-VTI11 either in the presence (black line) or absence of YKT62 (green line) and mixing each R-SYP4 family and either R-MEMB11 (yellow line) or R-VTI12 (red line) in the presence of YKT62. **D**. Maximum percentages of fusion of donor vesicles containing R-VTI11, R-VTI12 or R-MEMB11 with vesicles reconstituted with a SYP4 family protein were analyzed statistically and compared. Maximum percentage of fusion between R-SYP42 and either R-VTI11 or R-VTI12 showed a significant difference by *t*-test (*p* = 0.006, labeled with asterisk). Error bars represent standard deviation (n = 7).

To test this hypothesis, VTI11 was reconstituted into donor vesicles (Figure 2) containing fluorescent lipids, and proteoliposome lipid mixing assays were performed with acceptor vesicles containing each SYP4 family member and donor vesicles containing VTI11, all in the presence of YKT62. As shown in Figure 4, all SYP4 family proteins were able to drive rapid lipid mixing with donor vesicles containing VTI11, suggesting that the SYP4 family is also able to form functional SNARE complexes with VTI11. When the maximum percentage of lipid mixing (measured as NBD fluorescence) containing one SYP4 family member with vesicles containing either VTI11 or VTI12 was compared, SYP41 and SYP43 showed similar maximum percentages. However, SYP42 showed more lipid mixing with VTI11 than VTI12 by 5-10%. This difference was found to be statistically significant (*t*-test, p < 0.05), suggesting that VTI11 functions with SYP42 better than VTI12 *in vitro* (Figure 4B,D), although whether this has biological significance *in vivo* is unknown.

We have shown that both VTI11 and VTI12 can drive lipid mixing in combination with SYP4 family members (Figure 3 and 4). To test whether the lipid mixing is driven by the specific interaction between individual SYP4 and VTI1 family members rather than a general requirement that any SNARE can fulfill, MEMB11, a v-SNARE involved in endoplasmic reticulum to Golgi anterograde trafficking and fusion at the Arabidopsis *cis*-Golgi, was synthesized and incorporated into donor vesicles [36]. Little lipid mixing was seen between vesicles containing MEMB11 and vesicles containing SYP4 family SNAREs in the presence of YKT62, indicating that the reactions are specific for the tested SNAREs (Figure 4).

**Figure 5:**
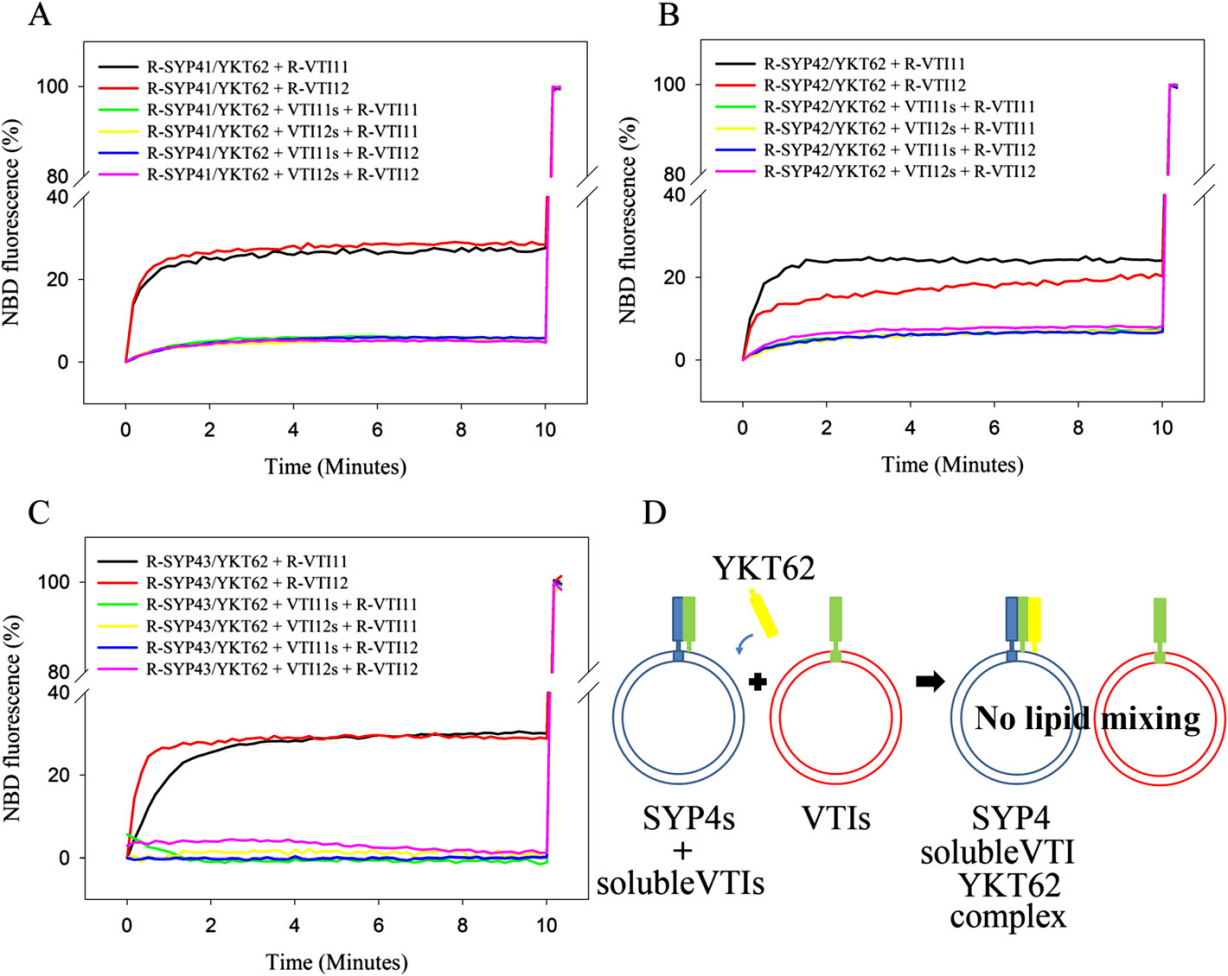
**SYP4 and VTI1 family-mediated fusion can be inhibited by soluble VTI11 or soluble VTI12.** Reconstituted vesicles containing either SYP41 **(A)**, SYP42 **(B)** or SYP43 **(C)** were mixed with YKT62 and either soluble VTI11 or soluble VTI12 for 30 min, followed by addition of either R-VTI11 or R-VTI12. The fluorescence did not increase significantly (less than 4%), indicating inhibition of the fusion reaction in the presence of soluble VTI11 or soluble VTI12. **D**. Schematic diagram of the assays in Figure 5.

As a further confirmation that the observed lipid mixing is specifically dependent on the VTI1 family, soluble fragments of VTI11 and VTI12 (VTI11s and VTI12s) were synthesized lacking the transmembrane domain. These soluble VTI11 or VTI12 fragments were mixed with YKT62 and acceptor vesicles containing one SYP4 family member, followed by incubation for 30 min before adding reconstituted donor vesicles and measuring lipid mixing (Figure 5D). The soluble VTI11 and VTI12 proteins inhibited the lipid mixing of vesicles containing their full length version. When cross inhibition (VTI11s with VTI12 and VTI12s with VTI11) was examined, the same inhibition was observed, presumably due to occupation of the entire available SYP4 family member by the soluble version of VTI11 or VTI12 proteins, thus preventing interaction with the full length version (Figure 5). Although SYP42 preferentially works with VTI11, VTI12s can still block VTI11 fusion. The disassembly of SNARE complexes requires NSF and SNAP [37]. Thus, the preformed SYP42 SNARE complex containing VTI12s and YKT62 cannot interact with VTI11, because the SYP42 SNARE complex is locked. These data indicate that the full-length proteins cannot displace the soluble fragments and confirm that lipid mixing is mediated by the interaction between SNAREs on the acceptor and donor vesicles.

These results demonstrate that individual SYP4 family members can interact with VTI11 during lipid mixing in addition to their interaction with VTI12. Inhibition by soluble VTI11 and VTI12 suggests that VTI11 and VTI12 may have similar abilities to drive membrane fusion. This implies that VTI11 can substitute for VTI12 in catalyzing membrane fusion via interaction with the same SYP4 family complexes to which VTI12 binds *in vivo* and provides a molecular explanation for the ability of VTI11 and VTI12 to partially compensate for each other in the respective mutants.

## Discussion

The Arabidopsis genome encodes three proteins that are members of the SYP4 family and four members of the VTI1 family. Previously, SYP41, VTI12 and YKT61/62 were found to be important components of the membrane fusion machinery at the Arabidopsis TGN [15,18]. Here, we demonstrate that other members of the SYP4 and VTI1 families are also able to drive rapid lipid mixing *in vitro*, and provide further evidence that family members have functional overlap. We use a lipid mixing assay based on dequenching of lipid fluorescence as a proxy for membrane fusion [12]. We have previously shown that SYP41-driven lipid mixing results from full fusion between the lipid bilayers, and not simply from hemi-fusion of the outer leaflets [15]. The other combinations of SNAREs used here show similar lipid mixing efficiencies, suggesting that they also result in full membrane fusion. It has been shown that lipid mixing precedes and is more efficient than content mixing during *in vitro* liposome fusion [38]; determination of whether the SNAREs used in this work can also lead to content mixing will require a specific assay for this process [39,40].

The degree of functional overlap between SYP4 family members has been controversial in the literature. One report indicated that each family member is essential for viability, suggesting that each SYP4 protein has a unique and essential function [22]. A second report, however, indicated substantial overlap in function, with no phenotype observed for single mutants and combinations of mutations required to observe trafficking defects [23]; a triple *syp41syp42syp43* mutation was lethal. The reason for these discrepancies is unknown, but our *in vitro* results indicate that all three SYP4 family members can drive lipid mixing with the same combinations of other SNAREs, providing support for functional overlap between family members.

It has been shown that SYP41 and SYP42 interact with only one VTI1 family protein, VTI12, *in vivo*, suggesting SYP41 and SYP42 are involved in membrane fusion with vesicles containing VTI12 as a v-SNARE [18]. This was confirmed by an *in vitro* lipid mixing assay using reconstituted SYP41 or SYP42 and VTI12 (Figure 3A,B). However, SYP42 showed an unexpected preference for VTI11 in the *in vitro* lipid mixing assay (Figure 4B). It is possible that the difference between immunoprecipitation results using Arabidopsis cell extract and lipid mixing assays using recombinant proteins could be caused by the distinct localization of VTI11 and VTI12 *in vivo*. VTI11 is hypothesized to function as a v-SNARE that targets vesicles containing the vacuolar sorting receptor VSR1 and ssVSD-containing vacuolar cargo from the TGN to the PVC, and VTI12 is thought to be involved in trafficking to storage vacuoles via recycling of vesicle trafficking components from the PVC to the TGN, although functional overlap between the two proteins is evident [24,27,29,41]. VTI11 localizes to the PVC and interacts with SYP21 and SYP51, while VTI12 localizes to the TGN [35]. The different localization of VTI11 versus VTI12 precludes the possibility of interaction of VTI11 with SYP4 family members at the TGN under normal conditions.

Although VTI11 and VTI12 seem to be involved in distinct trafficking pathways, they can compensate for loss of the other protein in *vti11* and *vti12* single mutants [26]. In addition, more VTI12 is expressed in a *vti11* mutant, presumably to make up for loss of VTI11, and a single amino acid substitution in VTI12 can partially suppress the phenotype of *vti11* mutants [29]. These results suggest that VTI1 family proteins can bind to non-cognate interaction partners when the other VTI1 family member is not available. This possibility was tested using *in vitro* lipid mixing assays, showing that VTI11 was able to mediate vesicle fusion by interacting with SYP4 family members. This result indicates that compensation by VTI11 in *vti12* mutants *in vivo* is likely to be via the ability to form a functional SNARE complex with the VTI12 binding partners. The inverse may also be true, that VTI12 may be able to interact with the normal VTI11 binding partners under the appropriate circumstances. *In vitro* fusion assays using recombinant SYP21 and SYP51 could provide further information about the redundant functions of VTI12 and VTI11. Our data support the hypothesis that the distinct subcellular localization of VTI11 and VTI12 *in vivo* is the primary determinant of fusion specificity of this family, rather than the innate biochemical properties of the proteins themselves.

## Conclusions

In conclusion, we have demonstrated that each SYP4 family member at the TGN, together with either VTI11 or VTI12, can drive proteoliposome lipid mixing *in vitro*. These data provide support for functional overlap between SYP4 family members [23] rather than distinct essential functions [22], an issue which has been controversial in the literature. They also indicate that VTI11 can substitute for VTI12 in driving lipid mixing with SYP41, suggesting that they have similar biochemical properties and that their specificity in different trafficking pathways *in vivo* is mainly due to their different subcellular localizations.

## Methods

### Plasmid construction

Full-length SYP42, SYP43, VTI11, MEMB11 and a soluble fragment of VTI11 (VTI11s, including amino acid sequence 1–200) were amplified by PCR using cDNAs generated from Arabidopsis seedlings and the following primers; SYP42 FP/RP, SYP43 FP/RP, VTI11 FP/RP, VTI11s FP/RP and MEMB11 FP/RP (Table 1). DNA fragments encoding SYP42 and SYP43 were digested using *EcoRI* and *XhoI*. DNA fragments encoding VTI11, VTI11s and MEMB11 were digested using *EcoRI* and *HindIII*, *BamHI* and *HindIII,* and *EcoRI* and *XhoI*, respectively, and ligated into the pET28a expression vector (Novagen, Madison, WI) digested using the same restriction enzymes.

**Table 1:** Primer sequences

**Gene**	**Direction**	**Sequence**
*SYP42*	Forward	5′-GGAATTCCATATGGCGACGAGGAATCGAACGACGGTG-3′
	Reverse	5′-CGCCTCGAGCTAAAACAAAATATTCTTAAGAATTAA-3′
*SYP43*	Forward	5′-GGAATTCCATATGGCGACTAGGAATCGTACGCTGTTG-3′
	Reverse	5′-CGCCTCGAGTCACAACAGAATCTCCTTGAGGATTAAGA-3′
*VTI11*	Forward	5′-GGAATTCCATATGAGTGACGTGTTTGATGGATATGAG-3′
	Reverse	5′-CCCAAGCTTTTACTTGGTGAGTTTGAAGTACAAGATG-3′
*VTI11s*	Forward	5′-GGATCCATGAGTGACGTGTTTGATGG-3′
	Reverse	5′-AAGCTTTTAGGTCCATTTGTTCTTGT-3′
*MEMB11*	Forward	5′-GAATTCATGGCGTCTGGTATCGTCGA-3′
	Reverse	5′- CTCGAGGGTTAGCGTGTCCATCTTATGA-3′

The restriction sites used for cloning are underlined.

### Protein expression and purification

Protein expression was performed as described in Chen et al. [15] with minor changes. SYP41, SYP42, SYP43, VTI11, VTI12, VTI11s, VTI12s, YKT62 and MEMB11 were expressed in *E. coli* strain BL21 (DE3) as N-terminal His_6_-tagged proteins. Ten ml of an overnight culture were transferred to 500 ml Luria-Burtani media with 50 μg/ml kanamycin and 2 mg/ml glucose. Cells were grown at 37°C until the OD600 reached 0.6. Isopropyl-β-D-thiogalactopyranoside was added to 0.5 mM final concentration to induce expression, and cells were incubated for 5 h at 16°C.

SYP41, VTI12s and YKT62 were purified according to Chen et al. [15], and SYP42, SYP43, VTI11, VTI12, VTI11s and MEMB11 were purified with minor changes in the washing and elution steps. SYP42 and SYP43 were eluted in elution buffer with 0.2% (v/v) Triton X-100 and VTI11s was purified in the same manner as VTI12s. VTI11, VTI12 and MEMB11 were washed sequentially using washing buffer I (50 mM NaH_2_PO_4_ (pH 8.0), 200 mM NaCl, 50 mM imidazole, 0.2% (v/v) Triton X-100), washing buffer II (50 mM Tris–HCl (pH 8.0), 300 mM NaCl, 50 mM imidazole, 0.2% (v/v) Triton X-100), washing buffer III (50 mM Tris–HCl (pH 8.0), 300 mM NaCl, 50 mM imidazole) and washing buffer IV (50 mM Tris–HCl (pH 8.0), 300 mM NaCl, 50 mM imidazole, 0.8% (w/v) n-octylglucoside), followed by elution using elution buffer (50 mM Tris–HCl (pH 8.0), 300 mM NaCl, 300 mM imidazole, 0.8% (w/v) n-octylglucoside).

### Preparation of lipid vesicles and membrane reconstitution

Preparation of lipid vesicles and reconstitution of proteins into vesicles were performed as described in Chen et al. [15] except for proteins inserted into donor vesicles (VTI11, VTI12 and MEMB11). Donor vesicles containing fluorescent dyes were mixed with VTI11, VTI12 or MEMB11 at a 1:200 protein-to-lipid molar ratio and incubated at 4°C for 30 minutes. The same volume of fusion assay buffer was added to the mixture, which was dialyzed overnight against fusion assay buffer with 4% (v/v) glycerol containing 1 g/l of Bio-beads SM-2 (Bio-Rad, Hercules, CA) to remove the trace amount of detergent. The reconstitution efficiency was analyzed by SDS-PAGE and staining with Coomassie Blue. The amount of protein in vesicles was compared with a known concentration of protein before reconstitution using densitometry (GS-800 Calibrated Densitometer, Bio-Rad, Hercules, CA) and Quantity one software (Bio-Rad, Hercules, CA).

### Removal of detergent from YKT62

The detergent in YKT62 samples was removed by adding 20 mg Bio-beads SM-2, followed by rocking at 4°C for 30 min. Removal of detergent was repeated three times, followed by dialysis as described above.

### Total lipid mixing assay

The donor and acceptor vesicles were mixed in a molar ratio of 1:9, and the same molar amount of YKT62 to SYP41, SYP42 or SYP43 was added to the mixture. The total lipid concentration was 0.5 mM and the total volume of the mixture was set to 100 μl. Fusion of donor vesicles with acceptor vesicles decreases quenching between rhodamine and NBD, measured as an increase in NBD fluorescence.

Fluorescence was measured at excitation and emission wavelengths of 465 and 530 nm, respectively. Fluorescence changes were recorded every 10 sec with a Varian Cary Eclipse model fluorescence spectrophotometer (Varian, Palo Alto, CA) with 2 mm path length at 25°C for 10 min. The maximum fluorescence intensity (MFI) was achieved by adding 1 μl of 10% (v/v) Triton X-100.

### Lipid mixing with VTI11s or VTI12s

To inhibit fusion between donor and acceptor vesicles, a soluble version of VTI11 or VTI12 was added to the acceptor vesicles and incubated for 30 min at room temperature. The incubated acceptor vesicles were used for the lipid mixing assay as described above.

## Abbreviations

SNARE: Soluble *N*-ethylmaleimide-sensitive factor attachment protein receptor; TGN: *Trans*-Golgi network; PVC: Prevacuolar compartment; SM: Sec1/Munc18; DOPC: 1,2-dioleoyl-sn-glycero-3-phosphocholine; POPG: 1-palmitoyl-2-oleoyl-sn-glycero-3-(phospho-rac-(1-glycerol)); POPE: 1-palmitoyl-2-oleoyl-sn-glycero-3-phospho-ethanolamine; NBD: *N*-(7-nitro-2,1,3,benzoxadiazol-4-yl); FRET: Fluorescence resonance energy transfer.

## Competing interests

The authors declare that they have no competing interests.

## Authors’ contributions

SJK performed the experiments, analyzed the results and drafted the manuscript. DCB conceived of the study, designed the experiments, analyzed the results and revised the manuscript. Both authors read and approved the final manuscript.
